# Optimization of Ultrasound Assisted Extraction of Bioactive Compounds from Apple Pomace

**DOI:** 10.3390/molecules26133783

**Published:** 2021-06-22

**Authors:** Itziar Egüés, Fabio Hernandez-Ramos, Iván Rivilla, Jalel Labidi

**Affiliations:** 1 Ma+D-Waste Recovery Engineering, Edificio Joxe Mari Korta—Avda Tolosa 72, 20018 Donostia-San Sebastián, Spain; itziar.egues@ehu.eus (I.E.); gerencia@valorizacion.net (I.R.); 2 Biorefinery Process Research Group Chemical and Environmental Engineering Department, University of the Basque Country (UPV/EHU), Plaza Europa, 1, 20018 Donostia-San Sebastian, Spain; fabio.hernandez@ehu.eus; 3 IKERBASQUE, Basque Foundation for Science, 48009 Bilbao, Spain

**Keywords:** ultrasound, optimization, apple pomace, antioxidant capacity

## Abstract

In the present work, the optimization of the extraction of antioxidant compounds from apple pomace using ultrasound technology as an environmentally friendly and intensification process was developed. Different sonication powers, extraction temperatures and extraction times were studied and their influence on extraction yield and characteristics of the extracted samples (total phenolic compounds, flavonoid content and antioxidant capacity) are presented. The elaborated experimental design and the analysis of Pareto and response surface diagrams allowed us to determine the optimal extraction conditions. The conditions that allow the maximum extraction of phenolic compounds were found at 20 min, 90 °C and 50% ultrasound amplitude. Nevertheless, at these conditions, the antioxidant capacity measured by DPPH decreased in the extracted samples.

## 1. Introduction

Waste generation and management is one of the main concerns in the industry. The waste generated in the food sector is generally rich in water and organic compounds and therefore these by-products can cause environmental problems. The manufacturers face additional costs for the adequate disposal or treatment of their residues. However, food waste could be a potential raw material for the extraction of valuable products. It is cheap and produced in large quantities as by-products. In this aspect, more comprehensive studies about its valorizations could lead to economic and environmental benefits [1].

Cider is an alcoholic beverage produced by the total or partial fermentation of apple juice. The manufacture of cider is carried out annually and Spain is the second largest producer in the European Union after the United Kingdom [2]. In 2017, the cider industry used more than 1,000,000 tons of apples during its production [2]. To produce cider, apples are harvested after the summer and then are pressed for several days. After pressing, apple pomace waste is generated, which is approximately 25–35% of the initial weight of the apples [3,4]. Then, the juice obtained is transferred to a vat where it undergoes spontaneous fermentation. As it can be seen, apple pomace is a waste generated in large volume during the cider manufacturing process. Therefore, new processes for its valorization are currently being investigated [4].

Apple pomace is composed of a mixture of pulp, epidermis, seeds and stems [5]. Besides water, this mixture contains mainly carbohydrates, phenolic compounds, proteins, fat, ash and crude fiber [6]. However, its composition varies according to the origin and the degree of maturation [7]. In the literature, the composition of the apple pomace covers a wide range of values (% on a dry basis): cellulose 7.2–43.6% [8], hemicellulose 4.26–29.90% [7,8], lignin 15.3–23.5%, pectins 3.5–14.32% [8], proteins 2.9–5.7% [8], lipids 1.20–3.9% [8] and ashes 1.47–1.66% [7]. Therefore, apple pomace is considered as a good source of fiber, pectin and phenolic compounds [9].

Some studies have used apple pomace as a source of high value-added products [4,9] for production of lactic acid and citric acid [8] and the production of sustainable biomaterials [10], among others. The extraction of bioactive compounds could be another interesting route. In fact, some studies indicated a possible adverse effect of the consumption of synthetic antioxidants; therefore, the extraction of antioxidant compounds from natural resources could be a promising route [11]. Recently, the potential application of apple pomace for antioxidant extraction was reported in several works [6,12,13,14,15]. Phenolic compounds like quercetin, catechin, phloridzin, gallic acid and chlorogenic acid can reduce chronic disease risk [13]. Leyva-Corral et al. (2016) [6] reported the presence of chlorogenic acid, epicatechin, rutin and phloridzin as phenolic compounds in apple pomace and their antioxidant capacity was assessed. Pingret et al. (2012) [12] also reported the chemical composition of polyphenols extracted from apple pomace, where catechin monomers and phenolic acids with antioxidant capacity were found. Ferrentino et al. (2018) [14] also reported the presence of phloridzin, epicatechin and quercetin and their antioxidant capacity was evaluated. In this context, the use of apple pomace as a source of phenolic compounds with antioxidant activity, particularly flavonoids, could be a promising route for its valorization, as they remain in the pulp during the cider production process [16,17].

In the literature, the extraction of phenols from apple pomace has been performed by different technologies. Among conventional methodologies, solid-liquid extraction, stirring and shaking [18] and the use of different organic solvents, namely methanol, acetone and ethanol [19,20] can be found. The optimization and intensification process are crucial steps for the development of a feasible process at industrial scale. In addition, the implementation of green technologies and the use of more environmentally friendly solvents are required. In this context, previous studies have shown that water could be a suitable extraction solvent able to extract phenolic compounds from apple pomace [17].

As a new environmentally friendly and intensification process, sonication-assisted extraction has gained attention. Ultrasound-assisted extraction is an effective and environmentally friendly method for bioactive compound extraction [21,22]. Its application could be an efficient alternative to traditional techniques, since it can improve the productivity, yield (as it increases the contact area between the solid and liquid phase) and selectivity of the process, reducing the cost of extraction operations [23,24]. Ultrasonic probes can be used for the application of ultrasound, where they can be immersed in the solution containing the sample for the extraction of the desired component. Ultrasonic probes have some advantages over an ultrasonic bath, because the ultrasound probe produces more energy than a conventional ultrasound bath. This technology can accelerate chemical reactions [25]. In recent studies, ultrasound technology has also gained attention for antioxidant extraction from apple pomace [12].

Therefore, the objective of this work was the optimization of the ultrasound-assisted extraction of antioxidant compounds from apple pomace using water as a solvent. The aim was to find the optimum conditions that allow a better extraction of valuable compounds such as antioxidants using environmentally friendly and intensification technologies. For that, we show the effect of time, temperature and sonication power on the extraction yield and the characteristics of the extracted samples (total phenolic compounds, flavonoids content and antioxidant capacity). For a better correlation and interpretation of the obtained results, different analyses and representations of Pareto and surface response diagrams were performed.

## 2. Results

### 2.1. Composition of Apple Pomace

The apple pomace we used had the following chemical composition (% on a dry basis): α-cellulose: 21 ± 0.5, hemicelluloses: 23 ± 0.5, klason lignin: 19 ± 0.4, pectins: 15.0 ± 0.5, proteins: 5.00 ± 0.3, lipids: 5.55 ± 0.3, and ashes content: 1.7 ± 0.1.

As described in the introduction section, the composition of the apple pomace is highly variable (% on a dry basis): cellulose 7.2–43.6% [8], hemicellulose 4.26–29.90% [7,8], lignin 15.3–23.5%, pectins 3.5–14.32% [8], proteins 2.9–5.7% [8], lipids 1.20–3.9% [8] and ashes 1.47–1.66% [7]. The characterization techniques used, as well as the origin of the apple pomace, may explain the variability of the results. In this work, the percentages found were in the range seen in the literature. Therefore, the apple pomace we used demonstrates its suitability for the extraction of biocomponents.

### 2.2. Effect of Extraction Time and Ultrasound Amplitude on TPC, TFC and AC

Table 1 summarizes the content of polyphenols (TPC), total flavonoids (TFC) and the antioxidant capacity (AC) per gram of dried apple pomace, extracted at 40 °C at different times and at different ultrasound amplitudes (50% and 70%).

As it can be seen, the TPC varies from 2.88 to 3.61 mg GAE/g and TFC ranges between 1.60–2.37 mg CE/g. The antioxidant capacity measured in the extracted liquors ranged from 0.92 to 1.39 mg TE/g.

Çam et al. (2010) [26] showed total phenolic levels between 2.23 and 6.18 mg GAE/g using ultrasound extractions and water as solvent. However, Candrawinata et al. (2015) [17] showed lower values of TPC between 0.83 and 1.26 mg GAE/g and antioxidant activity between 1.00 and 1.65 mg TE/g (by DPPH). In this work, the obtained experimental values showed acceptable levels.

On the other hand, Hernández-Carranza et al. (2016) [27] obtained a flavonoid content between 4.55 and 6.63 mg of CE/g of apple pomace, using conventional extraction methods (magnetic stirred hot plate and constant agitation of 3–12 h). The values obtained in this study (1.60 to 2.34 CE/g) were lower than those reported in literature. These differences could be affected by the extraction technique used, the extraction time, and the origin of the raw material used in the different studies.

**Figure 1:**
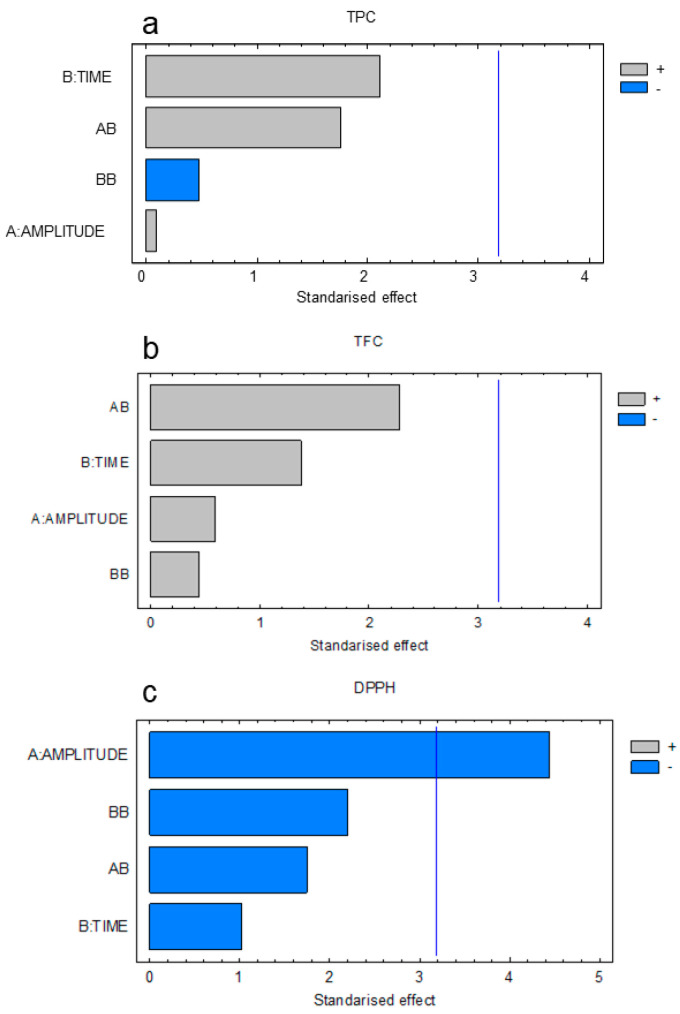
Standardized Pareto diagram of the screening for (**a**) TPC, (**b**) TFC, and (**c**) DPPH.

The adequacy of the results was verified by analysis of variance (ANOVA). The Pareto diagrams obtained in the ANOVA analysis are shown in Figure 1. As it can be noted, the effect of the amplitude affects the antioxidant activity measured by the DPPH assay. On the other hand, it should be noted that the TPC and TFC were not significantly affected by any of the variables studied.

This behavior was verified by studying the adequacy of the model through the study of the F-ratio whose significance can be evaluated using the *p*-value establishing a confidence level of 95%. Thus, a *p*-value under 0.05 implies that the studied effect significantly affects the response. The values of the F-ratio and *p*-values obtained using the Statgraphics software are shown in Table 2. As can be seen, only the independent variable amplitude had a significant effect on the antioxidant activity (DPPH), confirming the aforementioned.

Furthermore, Figure S1 (supplementary data) shows the impact of variables on different responses. According to the figures it can be concluded that the longer the extraction time, the more the responses (TPC, TFC, DPPH) increased, while increasing the amplitude decreased the properties of the extracts.

Considering these results, we established an amplitude of 50% to carry out further experimental design, as shown in Table 3.

### 2.3. Optimisation of the Extraction Conditions

Table 4 summarizes in detail the set of experiments obtained using the Statgraphic software. In addition, it also includes the experimental values corresponding to the dependent variables Y_TPC_, Y_TFC_, and Y_DPPH_, and the extraction yield.

Regression coefficients together with their corresponding significance levels according to Student’s *t*-test, as well as the determination coefficient (R^2^), fitted R^2^ and statistical significance (Fisher’s F test) are summarized in Table 5.

As shown in Table 5, the determination coefficients R^2^ obtained for each of the studied dependent variables Y_TPC_, Y_TFC_, and Y_DPPH_ were 0.97, 0.94 and 0.99, respectively, indicating the validity of the design. These high determination coefficient values indicate that a small number of the total variations remain unexplained, concretely 0.03%, 0.06%, and 0.01% for Y_TPC_, Y_TFC_, and Y_DPPH_, respectively. Such values, together with the high F values, indicate that the selected model is adequate to represent the selected variables and the model result is statistically representative.

**Figure 2:**
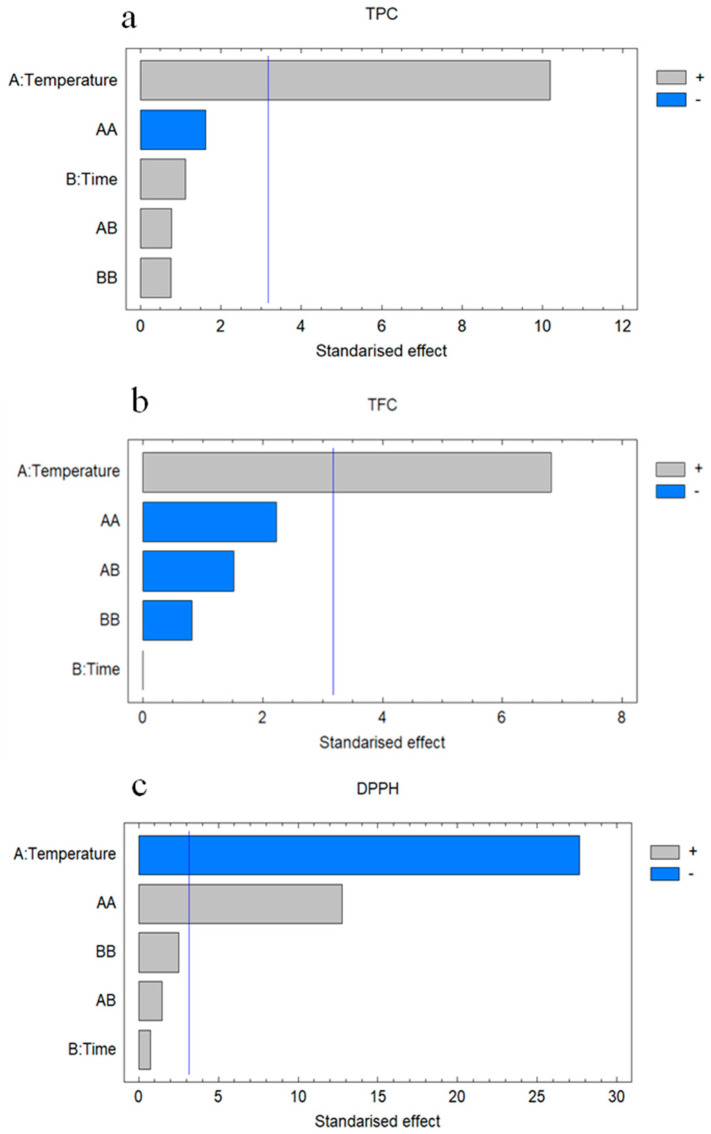
Standardized Pareto diagram for (**a**) TPC, (**b**) TFC, and (**c**) DPPH.

According to the regression coefficients reported in Table 5, as well as in the Pareto diagrams presented in Figure 2, temperature (X_1_) is the most independent variable that influences obtaining the phenolics, flavonoids and antioxidant capacity. On the other hand, it should be noted that the independent variable X_2_ (time) did not exhibit a significant influence over any of the dependent variables. Regarding the quadratics effects (X_12_ and X_22_), it should be noted that they only showed significant influence on the antioxidant capacity of the obtained extracts.

In addition, the extraction yield (Table 4) increased as the temperature increased, obtaining the maximum yield of 58.44% at a temperature of 90 °C, while the minimum yield of 24.8% was obtained at the lowest temperature (40 °C).

#### 2.3.1. Influence of the Studied Variables

**Figure 3:**
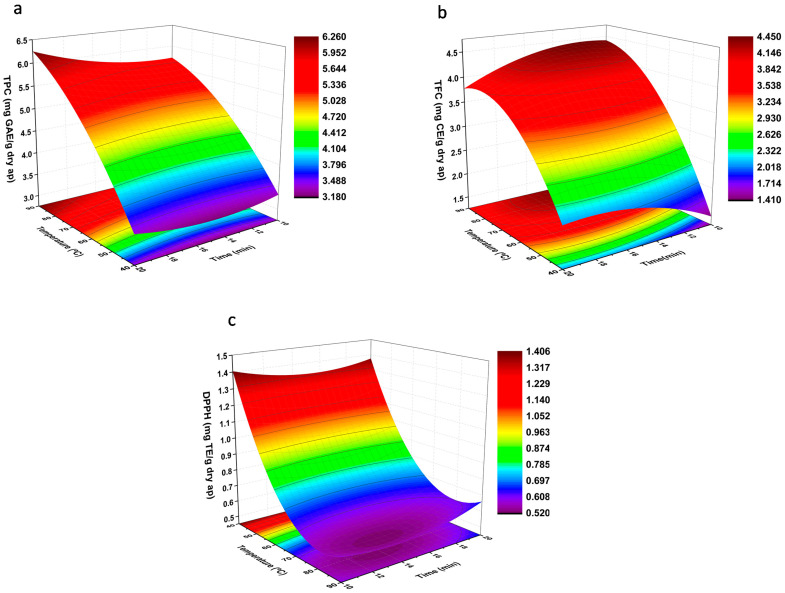
Response surface diagrams showing optimal extraction time conditions and temperature against (**a**) TPC, (**b**) TFC, and (**c**) antioxidant properties.

The interaction of the independent variables and their influence on the TPC, TFC, and DPPH are shown in Figure 3.

##### Total Phenolic Content (TPC)

Different research indicated that the extraction of phenols from food waste was improved with the temperature and time [12,27]; however, heat degradation may occur at high temperatures. In this study, the TPC was strongly affected by the effect of the temperature, while time exhibited a low effect as indicated by the regression coefficient corresponding to the Y_TPC_ dependent variable (Table 5).

Figure 3a shows the response surface corresponding to the TPC as a function of the independent variables X_1_ and X_2_. According to this figure, it can be appreciated that as the temperature (X_1_) increases, the TPC increases independently of the extraction time used. It is reported in the literature that the length of time for the extraction was also an important factor when the extraction is performed below the critical temperature [17]. In this work, the range studied (10, 15, 20 min) did not have strong influence.

The TPC content of the experiments were in the range of 3.10 to 6.02 (mg GAE/g) as shown in Table 4. The lowest value corresponded to the experiment 2 (40 °C and 15 min) while the highest value was obtained for the highest temperature and maximum extraction time in experiment 9 (90 °C, 20 min). Although time has little influence, it can be observed that at the maximum temperature of 90 °C and the longer extraction time, there was a slight improvement in the TPC content. This result is in agreement with Candrawinata et al. (2015) [17], who concluded that the TPC values increased with longer extraction times.

##### Total Flavonoid Content (TFC)

Similarly to TPC, the flavonoid content is mainly affected by the extraction temperature, while time has a low influence due to the low significance of its regression coefficient (Table 5). However, in this case, unlike TPC, the highest TFC value (4.59 mg CE/g) was obtained when the extraction time was 10 min (experiment 7). On the other hand, the lowest value was observed in experiment 1, at 40 °C and 10 min, as can be shown in Table 4 and Figure 3b.

##### Antioxidant Capacity (DPPH)

In Figure 3c, it is shown how temperature (X_1_) and time (X_2_) variables affect the antioxidant capacity of the extracts. As in the previous cases, time has no significant influence, and the antioxidant capacity is only affected by the effect of temperature. However, in this case, temperature has a negative effect on the obtained response, i.e., at low temperatures, the antioxidant capacity was higher. Thus, the highest antioxidant capacity is obtained for experiment 1 (1.39 mg TE/g), while at 90 °C and 15 min (experiment 8), the capacity is minimal (0.53 mg TE/g).

As it can be seen, the temperature significantly improved all responses except the DPPH values, i.e., at higher extraction temperature, higher TPC was obtained; however, the antioxidant capacity measured by DPPH was lower. These conclusions may be contradictory, since some studies have shown a correlation between the content of phenolic compounds and the antioxidant capacity [17,28]. Nevertheless, it is important to highlight that the determination of the total polyphenol content by the Folin–Ciocalteu method can generate an overestimation of the measurements since it can react with other reducing compounds, as sugars [29]. On the other hand, some studies have reported that the antioxidant capacity decreased with temperature, because exposure of phenolic compounds to high temperature can cause their degradation [30].

#### 2.3.2. Optimization of the Extraction Conditions and Model Validation

The objective of the optimization was to determine the best extraction conditions to provide the highest values of phenolic and flavonoid compounds and the greatest antioxidant capacity. For this purpose, the desirability function was used, employing the Statgraphics Centurion XVI software:

TPC = 4.86117 + 1.31392*T* + 0.1457*t* − 0.36155*T*^2^ + 0.12155*Tt* + 0.1697*t*^2^ (1)

TFC = 3.82718 + 1.17562*T* − 0.0005333*t* − 0.666217*T*^2^ − 0.32105*Tt* − 0.245167*t*^2^ (2)

DPPH = 0.64333 − 0.381667*T* + 0.01*Tt* + 0.305*T*^2^ + 0.025*Tt* − 0.06*t*^2^ (3)

The optimum conditions for the studied independent variables were 90 °C and the extraction time of 18.43 min. Under the cited optimum conditions, the estimated values were: TPC = 6.07 mg GAE/g, TFC = 4.00 mg CE/g and DPPH = 0.62 mg TE/g.

### 2.4. Relation between TPC and Antioxidant Capacity

**Figure 4:**
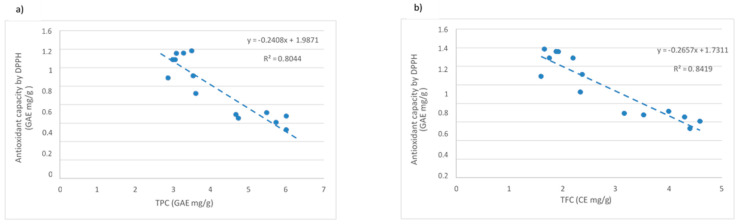
Correlation between (**a**) TPC and (**b**) flavonoid content with antioxidant activity tested by DPPH assay.

As commented before, there are studies that suggest the existence of a relationship between the total content of polyphenols and the antioxidant activity of the sample [17,28]. In Figure 4, the relationship between the content of phenolic compounds (Figure 4a) and flavonoids (Figure 4b) against antioxidant capacity determined by the DPPH method is shown. It can be observed that at higher concentrations of polyphenols and flavonoids, the antioxidant capacity measured by DPPH remained stable or decreased.

These results may be due to the fact that the quality of the extracted phenolics and flavonoids could be worse with the temperature and consequently, they could provide worse antioxidant capacity measured by DPPH. In addition, other reducing compounds could interfere in the Folin–Ciocalteu [29] and flavonoid tests. In order to observe the quality of the obtained samples, the antioxidant capacity of the samples is divided by the total phenols, and the results are shown in Table 6 (represented as Trolox equivalent/GAE). On the other hand, the radical inhibition capacity of the samples is also demonstrated.

As it can be seen, the reduction capacity shown by the compounds extracted from apple pomace at 40 °C and 50% amplitude was around 40%, where this value decreased until 23% at 65 °C and 90 °C extraction temperature. These values indicate that the highest percentage of reduction was registered in the samples extracted at lower temperature, which is in agreement with the previous obtained results. On the other hand, the antioxidant capacity of the commercial antioxidant compound Trolox was slightly higher, about 65%.

### 2.5. Sugars Characterization of the Extracted Samples

In Table 7, the sugar analysis and the obtained by-products in the extracted samples at different temperatures and extraction times is shown.

As described in the chemical composition of apple pomace, it has high amount of carbohydrate. In the literature, fructose is the highest simple sugar reported in apple pomace [13]. Glucose accounts for about 10 to 12% (dry weight basis). Apple pomace is also a high source of arabinose and rhamnose, which are components of biopolymers such as hemicellulose. In this study, the results showed the presence of glucose and fructose, in ranges of 1.35–1.47 g/L and 4.00–4.75 g/L, respectively. As by-products, citric acid and hydroxymethylfurfural were observed in smaller amounts. As it can be seen, with higher temperatures, the extraction of sugars was slightly higher. This effect could also explain the increment of the yield obtained at higher temperatures.

## 3. Materials and Methods

### 3.1. Raw Material

The apple pomace used in this study was provided by R. ZABALA cider producers (Gipuzkoa, Basque Country, Spain). The samples were collected after pressing the apple, which was then vacuum packed and stored at −40 °C. Then, in the laboratory, samples were dried at 50 °C for 72 h until a constant weight. The apple pomace was then ground and sieved to a particle size less than 250 µm and stored in a dry and cold place.

### 3.2. Characterization of the Apple Pomace

The raw material was characterized by klason lignin (TAPPI T222 om-98); α-cellulose [31] and hemicellulose. The moisture (at 100 °C) and ash content (at 550 °C) was evaluated by observing the weight loss until a constant value following the JAOAC 17, 68 (1934) and JAOAC 7, 132 (1923) food standards, respectively. Pectin extraction was carried out with the method described by Kumar et al. (2010) [32]. The proteins present in the sample were determined using elemental analysis for nitrogen quantification. For that, this content was multiplied by the universal factor of 6.25 [5].

### 3.3. Experimental Design of the Ultrasound Assisted Extraction

#### 3.3.1. Ultrasound Extraction Procedure

Ultrasound equipment (Vibra-Cell™ VC 750 W Ultrasonic Liquid Processor, Sonics & Materials, Church Hill Rd, Newtown, CT, USA) was used for the extractions. A glass container with a cooling jacket was used as recipient and as process temperature control. The container was connected to a bath equipped with a pump in order to recirculate the water at the desired temperature. This also allowed the temperature increments generated by the ultrasound process to be regulated. The optimum solid–liquid ratio was determined by taking into account the capacity of the ultrasound equipment and the liquid absorbing capacity of the apple pomace. Two grams of dried powder apple pomace were placed in the glass container with 80 mL of deionized water. Sonication was performed by immersing the ultrasonic probe to a depth of 1/3 of the height of the container. The extraction yield was measured by evaporating the extracted sample at 105 °C until a constant weight.

#### 3.3.2. Screening Study to Assess the Amplitude Settings

Prior to the experimental design, it was necessary to determine the optimal conditions of amplitude and time to maximize the extraction of the antioxidant compounds. To do this, a two-level factorial design was developed using the Statgraphics Centurion version XVI (Statpoint Technologies INC., Warrenton, VA, USA). In the screening study, two different amplitudes (50% and 70%) were studied using different extraction times (5, 10, 15 and 20 min) to evaluate their influence on TPC, TFC, and DPPH properties. Other variables such as the solid–liquid ratio (1:40, *w*/*v*), the type of solvent (water), the particle size (<250 µm), and the temperature (40 °C) were established as fixed factors. As a result of this preliminary study, the amplitude of 50% and times from 10 to 20 were selected.

#### 3.3.3. Experimental Design

After determining the appropriate amplitude of 50%, the influence of extraction temperature (65 °C and 90 °C) and extraction time (10, 15, 20 min) on TPC (mg GAE/g), TFC (mg CE/g) and DPPH (mg TE/g) properties were evaluated. For that, a response surface methodology (RSM) was employed. For this purpose, a three-level central design (3k), including a central point, was designed.

The data was fixed by employing a second-order polynomial equation (Equation (4)):

(4) $$y_{j}=\beta_{0}+\overset{k}{\underset{i=1}{\sum }}\beta_{i}X_{i}+\overset{k}{\underset{i=1}{\sum }}\beta_{ii}X_{i}^{2}+\sum^{\;}\overset{k}{\underset{i<j=1}{\sum }}\beta_{ij}X_{i}X_{j}+\varepsilon$$

where ***k*** corresponds to the number of factors (2), Y represent the dependent variables, namely, Y_TPC_ (Total phenolic content), Y_TFC_ (Total flavonoid content) and Y_DPPH_ (antioxidant capacity). ***β*_0_**, ***β_i_****, **β_ii_*** and ***β_ij_*** are the regression coefficients determined from the experimental results, employing the least-squares method. Finally, ***X_i_*** and ***X_j_*** are the normalized and dimensionless independent variables varying from −1 to 1 and ***ε*** is the experimental error. In order to validate the model, some parameters such as the lack of fit, the R^2^ (determination coefficient), the significance of the regression coefficients, and the F-test value acquired from the statistical analysis of variance (ANOVA) were evaluated. The model was validated through a triplicate of the experiment at the optimum point.

The experimental design as well as the optimum point were obtained employing the Statgraphics Centurion version XVI (Statpoint Technologies INC., Warrenton, VA, USA). The experimental data of the experimental design was adjusted through the regression analysis function of Microsoft Excel Add-In (Microsoft, Redmond, WA, USA).

### 3.4. Determination of the Total Content of Phenols and Flavonoids

#### 3.4.1. Total Phenolic Compounds (TPC)

The total polyphenolic content was measured spectrophotometrically following the Folin–Ciocalteu procedure, as described by Dudonné et al. (2009) [33], with some modifications. The liquor (0.3 mL; obtained after the treatment with ultrasound) was taken and mixed with 2.5 mL of the Folin–Ciocalteu reagent (previously diluted 1:10 with distilled water) and 2 mL of sodium carbonate (75 g/L in solution aqueous) was added. The mixtures were incubated at 50 °C in a hot bath for 5 min, and the absorbance was measured at 760 nm. A calibration curve was performed with gallic acid (0–300 mg/L, R^2^ = 0.994) and the results were expressed as milligrams of gallic acid equivalents (GAE) per gram of dry weight (mg GAE/g).

#### 3.4.2. Total Flavonoid Content (TFC)

The total content of flavonoids was determined spectrophotometrically following the method described by Lima et al. (2017) [34], with some modifications. The liquor (0.5 mL) was mixed with 2 mL of distilled water and 0.15 mL of sodium nitrite (5% in aqueous solution). The mixtures were incubated at room temperature for 5 min. Then, 0.15 mL of aluminum chloride hexahydrate (10%) was added and the solution was maintained for another 5 min. Finally, 1 mL of sodium hydroxide was incorporated, the total volume was adjusted to 5 mL with distilled water, and the absorbance at 510 nm was measured. A calibration curve with catechin (0–300 mg/L, R^2^ = 0.999) was prepared to transform the absorbance values obtained into the total content of flavonoids and the results were expressed as milligrams of catechin equivalents (CE) per gram of dry weight (mg CE/g).

### 3.5. Antioxidant Activity (AC)

The antioxidant capacity was determined spectrophotometrically following the method described by Gullón et al. (2017) [35] with some modifications. The sample (0.3 mL) was added to 3 mL of a fresh DPPH solution (0.06 mM in 96% ethanol, *v*/*v*). After the incubation of the samples in the dark at room temperature for 15 min, the absorbance at λ = 515 nm was measured. A calibration curve with Trolox (0–500 mg/L, R^2^ = 0.997) was prepared and the results were expressed as milligrams of Trolox equivalents (TE) per gram of dry weight (mg TE/g).

### 3.6. Radical Inhibition Capacity

The radical inhibition capacity of the samples was calculated as described by Dudonné et al. (2009) [33] following the formula (Equation (5)):

% inhibition = (AB − AE)/AB × 100(5)

where AB is the absorbance of the blank and AE is the absorbance of the apple pomace extract.

### 3.7. Sugars Characterization

The sugar quantification in extracted liquors was quantitatively determined in a high performance liquid chromatograph ((HPLC) Jasco LC Net II/ADC (column oven and quaternary gradient pump), (Jasco Corporation, Hachioji, Tokyo, Japan) equipped with a refractive index detector and a photodiode array detector. We used 0.005 N H_2_SO_4_ prepared with 100% deionised and degassed water as mobile phase (0.35 mL/min flow, 40 °C and injection volume 20 µL). High purity of different sugars such as glucose, fructose, xylose, arabinose, and rhamnose, and other by-products such as formic acid, citric acid, ethanol, lactic acid, furfural and hydroxymethylfurfural (HMF) were used for the calibration curve.

## 4. Conclusions

In this work, the extraction of antioxidants was carried out from apple pomace, using an environmentally friendly technology assisted by ultrasound and water as solvent. Furthermore, the optimization of the operative conditions was developed. The maximum extraction of phenols was detected at 90 °C and at 20 min using 50% of ultrasound amplitude. The results showed that at high extraction temperature, the content of polyphenolic and flavonoids content was higher. In contrast, the antioxidant capacity measured by the DPPH method was decreased where the highest antioxidant capacity was obtained at low temperatures and at short extraction times. This study is a starting point for future studies, where investigation should focus on the characterization, purification and application of the extracted biocompounds. This work contributes to the valorization of apple pomace using environmentally friendly intensification technologies and the optimization of the different variables for antioxidant extraction.

## Figures and Tables

**Table 1:** Content of phenolic compounds (TPC), total flavonoids (TFC) and antioxidant capacity (using DPPH assays) for different extraction times and ultrasound power.

Amplitude (%)	Time (min)	TPC (mg GAE/g *)	TFC (mg CE/g *)	AC (DPPH) (mg TE/g *)
50	5	3.07 ± 0.16	2.20 ± 1.12	1.29 ± 0.32
	10	3.51 ± 0.22	1.66 ± 0.21	1.39 ± 0.13
	15	3.10 ± 0.70	1.92 ± 0.12	1.36 ± 0.27
	20	3.29 ± 0.43	1.88 ± 0.15	1.36 ± 0.13
70	5	2.88 ± 0.14	1.60 ± 0.30	1.09 ± 0.27
	10	3.00 ± 0.30	1.76 ± 0.18	1.29 ± 0.20
	15	3.54 ± 0.33	2.37 ± 0.31	1.11 ± 0.14
	20	3.61 ± 0.57	2.34 ± 0.17	0.92 ± 0.23

* Values presented are the average of triplicate measurements.

**Table 2:** Adequacy of the model through the analysis of F-ratio and *p*-value.

	TPC		TFC		DPPH	
	F-Ratio	*p*-Value	F-Ratio	*p*-Value	F-Ratio	*p*-Value
Amplitude (A)	0.01	0.9303	0.35	0.5951	19.72	0.0212
Time (B)	4.45	0.1255	1.9	0.2622	1.05	0.3816
AB	3.08	0.1775	5.21	0.1067	3.05	0.1793
BB	0.23	0.6674	0.2	0.6844	4.83	0.1154

**Table 3:** Experimental variables of the experimental design.

Variable	Designation	Units	Nomenclature	Value
Fixed	Amplitude	(%)		50
	Solid-liquid ratio	(*w*/*v*)		1:40
	Solvent		water	
	Particle size	µm		<250
Independent	Temperature	(°C)	Temp	65–90
	Time	(min)	t	10–20
Dependent	Total phenolic content	(mg GAE/g)	TPC	Y_TPC_
	Total flavonoid content	(mg CE/g)	TFC	Y_TFC_
	Antioxidant capacity	(mg TE/g)	DPPH	Y_DPPH_

**Table 4:** Set of experiments and experimental results.

Exp.	Independent Variables		Normalized Variables		Dependent Variables			Yield (%)
	Temperature(°C)	Time (min)	X_1_	X_2_	Y_TPC_ (mg GAE/g *)	Y_TFC_ (mg CE/g *)	Y_DPPH_ (mg TE/g *)	
1	40	10	−1	−1	3.51 ± 0.22	1.66 ± 0.21	1.39 ± 0.13	24.8 ± 0.07
2	40	15	−1	0	3.10 ± 0.70	1.92 ± 0.12	1.36 ± 0.27	28.6 ± 0.10
3	40	20	−1	1	3.29 ± 0.44	1.88 ± 0.15	1.36 ± 0.13	29.5 ± 1.10
4	65	10	0	−1	4.68 ± 0.36	3.16 ±1.08	0.69 ± 0.38	39.5 ± 0.05
5	65	15	0	0	4.74 ± 1.84	4.30 ± 1.05	0.65 ± 0.27	55.1 ± 0.10
6	65	20	0	1	5.50 ± 0.95	4.00 ± 1.87	0.71 ± 0.16	50.6 ± 1.03
7	90	10	1	−1	5.75 ± 0.72	4.59 ± 0.23	0.61 ± 0.11	50.6 ± 0.05
8	90	15	1	0	6.02 ± 0.28	4.40 ± 0.72	0.53 ± 0.17	58.4 ± 1.15
9	90	20	1	1	6.02 ± 0.29	3.53 ± 0.82	0.68 ± 0.14	38.7 ± 1.50

* Values presented are the average of triplicate measurements.

**Table 5:** Regression coefficients and statistical parameters of the experimental design.

Regresion Coefficients	Y_TPC_	Y_TFC_	Y_DPPH_
b_0_	4.86 ^a^	3.83 ^a^	0.64 ^a^
b_1_	1.31 ^a^	1.18 ^a^	−0.38 ^a^
b_2_	0.15	0.00	0.01
b_12_	0.12	−0.32	0.02
b_11_	−0.36	−0.67	0.31 ^a^
b_22_	0.17	−0.25	0.06 ^b^
R^2^	0.9731	0.9400	0.9900
R^2^ fitted	0.9284	0.8600	0.9900
F-exp	21.7720	10.8585	187.0543
F-critical	0.0145	0.0387	0.0006
Significance level	98.54	96.12	99.94

^a^ Significant coefficients at the 99% confidence level. ^b^ Significant coefficients from the 90% to 95% confidence level.

**Table 6:** The quality (represented as Trolox equivalent/GAE) and % of inhibition capacity of apple pomace extracted samples against the radical DPPH (after 15 min) and commercial antioxidant Trolox at 0.05 g/L of concentration.

Time (min)	Temperature (°C)	Quality (TE/GAE)	Inhibition (%)
10		0.40	43.84
15	40	0.44	42.94
20		0.41	42.99
10		0.15	22.62
15	65	0.14	21.22
20		0.13	23.35
10		0.11	20.52
15	90	0.09	17.70
20		0.011	22.97
		Trolox	65.27

**Table 7:** Sugar and by-product analysis of the obtained samples at different temperatures and extraction times (at 50% of amplitude).

Temperature (°C)	Time (min)	Glucose (ppm)	Fructose (ppm)	Citric Acid (ppm)	HMF (ppm)
40	10	1353.92	4217.60	94.83	2.24
	15	1287.18	4000.80	98.69	10.13
	20	1020.23	3215.00	58.07	-
65	10	1383.80	4431.30	51.48	449.40
	15	1398.80	4494.20	24.03	-
	20	1467.30	4749.00	21.03	-
90	10	1475.80	4754.30	-	-
	15	1430.80	4524.80	5.75	-
	20	1423.80	4609.60	-	-

## Data Availability

Data is contained within the article.

## Supplementary Materials

The following are available online. Figure S1. Screening study response surface plots; Figure S2. Preparation of the apple pomace for further antioxidants extraction, (a) Frozen and vacuum packed, (b) Oven dried, (c) Grinding and sieving; Figure S3. Ultrasonic equipment and thermostatic bath used in the experiments.
