# Supplementary material for: Optimization of Ultrasound Assisted Extraction of Bioactive Compounds from Apple Pomace
Source: Molecules. 2021 Jun 22;26(13):3783. doi: 10.3390/molecules26133783 (PMC8270251; doi:10.3390/molecules26133783)
Supplement: Supplementary file 1 [file molecules-26-03783-s001.zip › molecules-1226819-SI.pdf]

SUPPLEMENTARY MATERIAL

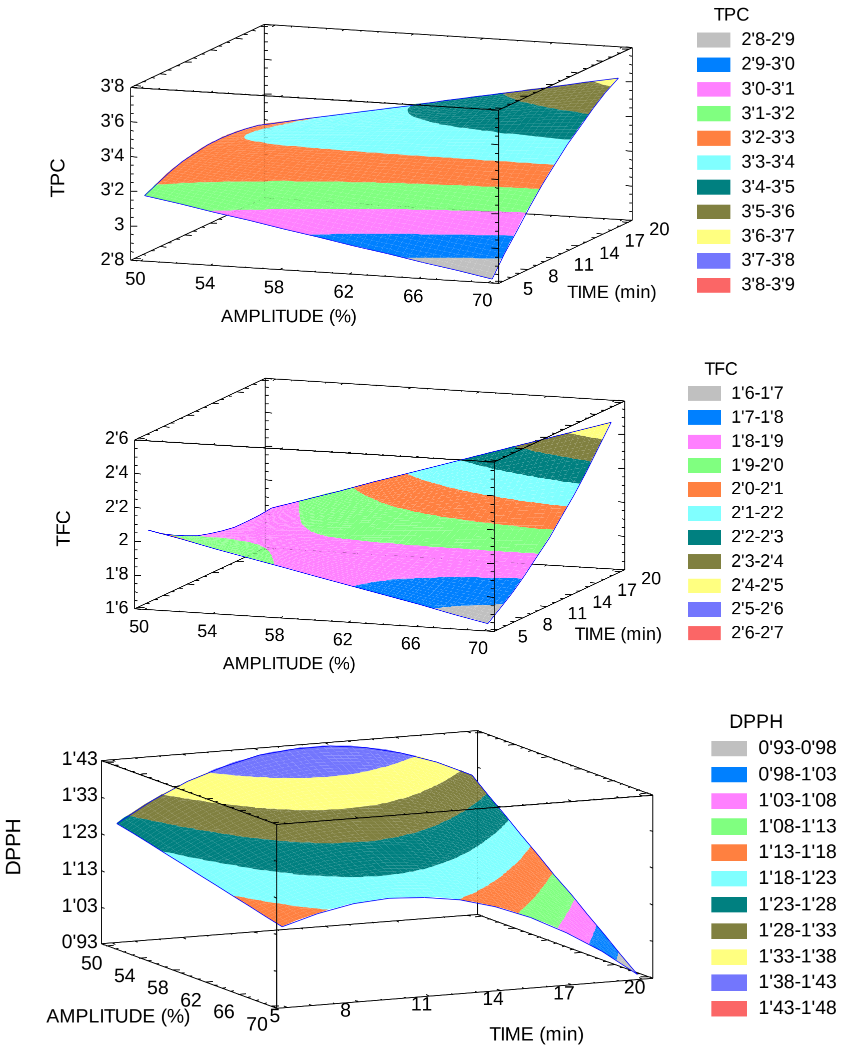

Figure S1. Screening study response surface plots

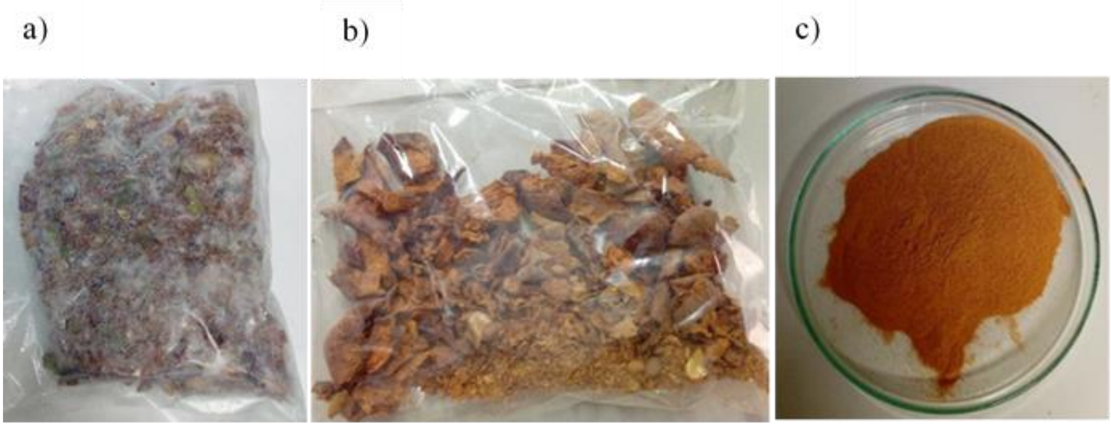

Figure S2. Preparation of the apple pomace for further antioxidants extraction a) Frozen and vacuum packed b) Oven dried c) Grinding and sieving

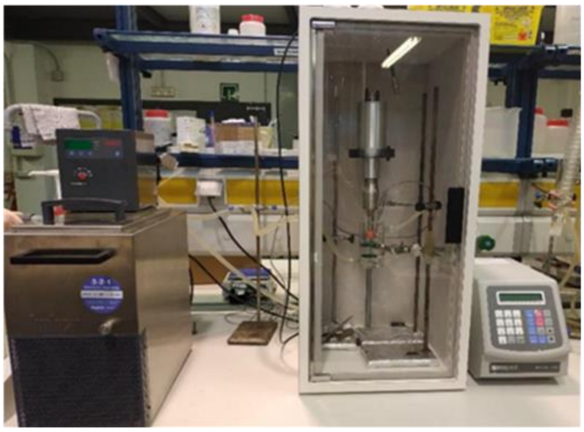

Figure S3. Ultrasonic equipment and thermostatic bath used in the experiments
